# Comparison of XEN45 Gel Stent Outcomes in Glaucoma: Ab Externo Open-Conjunctiva Approach with Ologen vs. Ab Interno Closed-Conjunctiva Approach

**DOI:** 10.3390/jcm14134426

**Published:** 2025-06-21

**Authors:** Sean J. Jin, Sharon Y. Kim, Jared Tallo, Harkaran S. Rana, Sorana Raiciulescu, Morohunranti O. Oguntoye-Ouma, Won I. Kim

**Affiliations:** 1 Department of Ophthalmology, San Antonio Military Medical Center, San Antonio, TX 78234, USA; 2 School of Medicine, Uniformed Services University, Bethesda, MD 20814, USA; sharkim7@gmail.com; 3 Department of Ophthalmology, Naval Medical Center San Diego, San Diego, CA 92134, USA; jared.tallo@gmail.com; 4 Department of Ophthalmology, Walter Reed National Military Medical Center, Bethesda, MD 20814, USA; rana.harkaran@gmail.com (H.S.R.); morohunranti.oguntoye-ouma.mil@health.mil (M.O.O.-O.); 5 Department of Preventive Medicine and Biostatistics, Uniformed Services University, Bethesda, MD 20814, USA; sorana.raiciulescu@usuhs.edu; 6 Eye Doctors of Washington, Chevy Chase, MD 20815, USA; wonkim74@gmail.com

**Keywords:** XEN45 gel stent, ab externo, ab interno, Ologen collagen matrix, Minimally invasive glaucoma surgery (MIGS), glaucoma surgery

## Abstract

**Background/Objectives:** This study evaluated the efficacy and safety of the ab externo open-conjunctiva (AEO) approach with adjunctive Ologen collagen matrix (OCM) compared to ab interno closed-conjunctiva (AIC) techniques for XEN45 gel stent implantation in patients with refractory open-angle glaucoma. The goal was to determine whether the AEO with OCM approach offers advantages in intraocular pressure (IOP) control and postoperative outcomes. **Methods:** A retrospective, comparative case series was conducted on 76 eyes from 76 patients with open-angle glaucoma who underwent XEN45 implantation between 2017 and 2022 at a single tertiary center. The patients were divided into Group 1 (AEO with OCM, n = 47) and Group 2 (AIC, n = 29). Postoperative IOP, the number of glaucoma medications, surgical complications, bleb revisions, and failure rates were recorded over 12 months. The AEO technique, supported by OCM, was assessed for its potential to reduce postoperative fibrosis and improve long-term outcomes. **Results:** Both groups experienced significant IOP reductions over time compared to baseline. However, Group 1 had superior outcomes, requiring fewer glaucoma medications postoperatively (*p* < 0.05), and demonstrated lower rates of complications (10.6% vs. 31.0%, *p* = 0.026) and bleb revisions (8.5% vs. 34.5%, *p* = 0.005). Kaplan–Meier survival analysis showed significantly greater cumulative surgical success in Group 1 compared to Group 2 (*p* < 0.001). **Conclusions:** The AEO with OCM approach to XEN45 implantation may provide improved safety and efficacy compared to the AIC approach. It appears to be beneficial in minimizing postoperative fibrosis, reducing the medication burden, and lowering complication and failure rates. Prospective randomized trials are needed to validate these findings.

## 1. Introduction

Glaucoma, an optic neuropathy, remains a significant contributor to irreversible vision loss and blindness globally [1]. Elevated intraocular pressure (IOP) stands as the sole modifiable risk factor among the myriad factors implicated in optic nerve damage [2]. Primary treatment methods include IOP-lowering medications, laser procedures, and surgery. Trabeculectomy, established in 1968, remains the gold-standard incisional surgical procedure for glaucoma treatment; however, it carries a notable complication rate of up to 57%, including hypotony, bleb leaks, choroidal effusion, endophthalmitis, and bleb-related infections [3,4].

In recent years, minimally invasive glaucoma surgery (MIGS) has emerged as a promising alternative to traditional incisional procedures [5]. One such innovation is the XEN45 gel stent (XGS), designed to provide a safer and less invasive means of lowering IOP [6,7]. The XGS, a 6 mm hydrophilic tube, facilitates aqueous humor drainage to the subconjunctival space, offering comparable efficacy and reduced complications compared to trabeculectomy within the first year post-surgery [8]. Its introduction has sparked interest in exploring different surgical techniques for optimal implantation, including ab interno closed-conjunctiva (AIC), ab interno open-conjunctiva (AIO), ab externo closed-conjunctiva (AEC), and ab externo open-conjunctiva (AEO) techniques [9]. AIC is currently the only Food and Drug Administration (FDA)-approved approach, requiring corneal incisions to insert the XGS.

A debate revolves around the optimal method for XGS implantation. The ab externo approach, particularly the open-conjunctiva technique, has garnered attention due to its potential benefits, including easier teaching, lower procedural costs due to reduced reliance on specialized instrumentation and reduced operating time, improved visualization during surgery, better control over bleb morphology and aqueous outflow, avoidance of corneal incisions, and overall improved prospects for long-term IOP control [10,11,12,13,14]. Conversely, the closed-conjunctiva approach offers simplicity and familiarity but may lack the advantages of the open approach. A recent study demonstrated that AEO led to significantly lower postoperative IOP compared with AIC, which was the first time a significant difference in IOP reduction was observed between these two procedures [15]. However, another study found no statistical difference in outcomes when comparing the AEO and AIC approaches, although both approaches were shown to be safe and efficacious [16].

Amid the gaps in knowledge and the variability in the findings, our study aimed to assess the efficacy of the ab externo open-conjunctiva approach, specifically when enhanced with adjunctive Ologen collagen matrix (OCM), compared to the FDA-approved AIC approach to XGS implantation. OCM, placed between the conjunctiva and the episcleral space, serves to prevent postoperative fibrosis and acts as a reservoir by absorbing aqueous humor into its porous structure [17,18]. While several studies have analyzed the effects of Ologen in other glaucoma surgeries, the utilization of Ologen for XGS implantation is still controversial considering the paucity of studies and the inconsistent results. Specifically, one study reported significant decreases in IOP and medication usage, alongside improved management of conjunctival fibrosis and a reduced need for postoperative interventions, with OCM augmentation [19]. However, another study utilizing an ab externo technique found no significant differences in surgical outcomes between XGS implantation with and without OCM augmentation [20].

To our knowledge, no study has directly compared the efficacy of AEO enhanced with OCM and the AIC approach to XGS implantation. We hypothesized that augmenting the AEO technique with OCM would result in more significant decreases in IOP, medication usage, and complication rates compared to the AIC approach. Ultimately, by evaluating the potential clinical advantages of the AEO approach enhanced with OCM over AIC, we aimed to inform surgical decision-making and enhance the efficacy and safety of glaucoma interventions.

## 2. Materials and Methods

### 2.1. Design

This study was a single-center, retrospective, comparative case series of 76 patients who underwent XEN45 gel stent placement between 2017 and 2022, with follow-ups ranging from 1 to 12 months, at the Walter Reed National Military Medical Center (WRNMMC). The study protocol was reviewed and deemed exempt by the WRNMMC Institutional Review Board (WRNMMC-EDO-2020-0546), and it complied with all Health Insurance Portability and Accountability Act (HIPAA) requirements. 

The patients were identified in a surgical database, and their data were retrieved from their electronic medical records. The inclusion criteria encompassed all adult patients (≥18 years) who underwent XEN45 gel stent implantation with AIC or AEO with OCM during the study period. Pediatric patients were excluded. Additional exclusion criteria included insufficient follow-up, defined as fewer than two postoperative visits with documented IOP and medication statuses. The recorded patient information included demographic variables such as age, gender, diagnosis, and the laterality of the eye undergoing surgery. The preoperative data encompassed visual acuity, IOP, the number of glaucoma medications, the extent of visual field loss, the pretreatment maximum IOP, and prior glaucoma procedures. The postoperative data included IOP, the medication burden, complications, revisions, surgical failures, and any additional glaucoma interventions. To assess the medication burden, each active IOP-lowering agent was counted as one medication, whether delivered as a single drop or as part of a fixed combination. For example, timolol–dorzolamide was counted as two medications. Oral agents such as acetazolamide were counted as one medication.

The primary outcomes were IOP reduction, the number of IOP-lowering medications, and cumulative success probabilities derived from Kaplan–Meier (KM) analyses. Secondary outcomes included postoperative complications, revisions, and surgical failure. For the KM survival analysis, surgical failure was defined as (1) having an IOP ≤ 5 mmHg or >18 mmHg on two consecutive visits after baseline or (2) the number of IOP-lowering medications being > 0 at any point during the follow-up visits. Postoperative complications included intraocular pressure (IOP) elevation > 30 mmHg, hyphema, iris obstruction of the stent, rebound iritis, wound leak, choroidal effusions, hypotony maculopathy, corneal decompensation, symptomatic bleb extension, cystoid macular edema, and corneal abrasion. Revisions were defined as transconjunctival bleb needling procedures. To account for the prior surgical status, surgical eyes were defined as those that had undergone at least one prior incisional glaucoma surgery, including trabeculectomy, installing glaucoma drainage devices, or microinvasive glaucoma surgery procedures such as the iStent or a prior XEN45 implantation. Eyes that had only undergone non-incisional procedures—such as laser peripheral iridotomy, selective laser trabeculoplasty, argon laser trabeculoplasty, or cataract extraction without concurrent glaucoma surgery—were not considered to have undergone a prior surgery.

Data points were recorded at specific intervals: postoperative day 1, week 1, weeks 2 to 3, months 1 to 2, months 3 to 4, months 5 to 8, and months 9 to 12.

### 2.2. Surgical Techniques

The surgeries were conducted by a single surgeon (W.I.K.) and were carried out using topical anesthesia. Group 1 consisted of patients who underwent AEO implantation of an XGS. Group 2 comprised patients who underwent one of two ab interno closed-conjunctiva techniques: (1) the standard ab interno closed technique and (2) an ab interno technique with pneumo-dissection and visco-expansion. The two AIC groups were combined for analysis based on a methodological precedent established by Ruda et al. in 2023, as well as considerations of the limited sample size [21]. The specific surgical techniques utilized are outlined below.

*Group 1: Ab externo open-conjunctival technique (AEO) (n = 47):* Tetracaine was applied for topical anesthesia. A 6 mm, fornix-based conjunctival flap was created, followed by infusion of 2% lidocaine into the sub-Tenon’s space for anesthesia. The XGS was implanted via an ab externo approach, entering the sclera 2 mm posterior to the limbus. A 12 mm by 1 mm disk of Ologen collagen matrix was folded in half and placed into the subconjunctival sub-Tenon’s space. The folded Ologen sheet was placed under and over the XGS to envelop it. The conjunctival peritomy was closed using an 8-0 Vicryl suture. Then, 0.1 to 0.15 mL of mitomycin C, with a concentration ranging from 0.2 to 0.4 mg/mL, was injected into the superior subconjunctival space.

*Group 2: Standard ab interno closed-conjunctiva technique (n = 18):* Mitomycin C, with a concentration ranging from 0.2 to 0.4 mg/mL, was injected superiorly into the subconjunctival space using a 30-gauge needle. A paracentesis port was created nasally with a sideport blade, followed by an injection of Methylparaben-free (MPF) 1% lidocaine and Miochol into the anterior chamber. The anterior chamber was filled with an ophthalmic viscoelastic device, and a 1.8 mm clear corneal incision was made inferotemporally. The XGS was implanted via an ab interno approach through the corneal incision under gonioscopic visualization. Residual viscoelastic material was irrigated out, and the anterior chamber was reformed with a balanced salt solution before suturing the clear corneal wound with a single 10-0 nylon interrupted suture.

*Group 2: Ab interno closed-conjunctival pneumo-dissection and visco-expansion technique (n = 11):* This technique was similar to the standard technique described above, except that air was introduced into the subconjunctival space via a 30-gauge needle approximately 6 mm posterior to the limbus. Subsequently, the pneumo-dissected space was further expanded with an ophthalmic viscoelastic device. The XGS was then delivered as described above into the supra-Tenon capsule space created by the pneumo-dissection and the viscoelastic device, allowing for separation of the conjunctiva and the Tenon capsule to form a supra-Tenon capsule pocket for stent implantation.

### 2.3. Statistical Analysis

Data analysis was conducted using commonly available software (Microsoft Excel and IBM SPSS Version 29). All tests were two-tailed, with statistical significance set at *p* < 0.05. Continuous variables were summarized as means with standard deviations or 95% confidence intervals, while categorical variables were expressed as frequencies and percentages.

Demographic variables were compared between the groups using the chi-square test. To analyze the trajectory of IOP over time and the effect of prior surgery, a linear mixed model was employed. This model was chosen to account for missing data at later postoperative time points and to allow pairwise comparisons between baseline and postoperative IOPs. The reported means are model-adjusted estimates.

Postoperative glaucoma medication use was analyzed using non-parametric methods due to the zero-inflated distribution of the data. The Mann–Whitney U test was used to compare the number of medications between the surgical groups at each time point, while the Wilcoxon signed-rank test was used for within-group comparisons between the baseline and postoperative values. These results are presented as medians, interquartile ranges (IQRs), and observed minimums (min) and maximums (max).

The complication rates and bleb revisions were compared between the groups using the chi-square test. Kaplan–Meier survival analysis was performed to estimate the time to surgical failure, defined as an intraocular pressure (IOP) ≤ 5 mmHg or >18 mmHg without the use of IOP-lowering medications on two consecutive follow-up visits or loss of light perception. The patients were censored at the time of failure, the last available follow-up, or at 12 months. The survival distributions were compared between the groups using the log-rank (Mantel–Cox) test.

## 3. Results

### 3.1. Baseline Clinical Data

This study included 76 eyes of 76 patients who underwent XEN45 gel stent implantation. Table 1 displays the preoperative clinical data and demographic characteristics of the study cohort. All patients had some form of open-angle glaucoma. At baseline, Group 1 had a mean visual field loss of −13.67 ± 7.81 dB and a mean preoperative IOP of 24.91 ± 6.75 mmHg on a median of 4.00 (min–max: 2–8) medications. Group 2 had a mean visual field loss of −12.46 ± 9.71 dB and a mean preoperative IOP of 22.76 ± 7.48 mmHg on a median of 4.00 (2–5) medications.

In total, 14 patients in Group 1 (30%) and 5 patients in Group 2 (17%) had a history of prior incisional glaucoma surgery, with no statistically significant difference between the groups. Previous surgeries were noted as follows: CyPass, iStent, trabeculectomy, Trabectome, gonioscopy-assisted transluminal trabeculotomy, Kahook Dual Blade, endoscopic cyclophotocoagulation, Baerveldt glaucoma implant, Ahmed glaucoma valve, and prior XEN45 gel stent implantation.

### 3.2. Follow-Up Data

Outcomes were recorded at each follow-up visit from postoperative day 1 to 12 months. The sample sizes of the patients at each postoperative time point are presented in Table S1 in the Supplementary Materials.

For Group 1, the mean preoperative IOP of 24.91 ± 6.75 mmHg dropped to 12.67 ± 4.05 mmHg on 0.61 ± 1.02 medications at the last follow-up. For Group 2, the mean preoperative IOP of 24.18 ± 7.75 mmHg decreased to 12.05 ± 2.46 mmHg on 1.91 ± 1.54 medications at the last follow-up. The IOP was significantly lower after XGS implantation in both Group 1 and 2 at all postoperative time points compared with the preoperative levels in all eyes (*p* < 0.001; Table 2). There were no postoperative time points after XGS implantation at which the IOPs were significantly different between the two groups (Table 2). However, the mean number of medications was significantly lower compared with baseline at each postoperative time point for both groups (*p* < 0.001). Group 1 (AEO with OCM) showed a significantly lower median medication requirement compared to Group 2 (AIC) at specific time points: at 1 week (median: 0.0 [min 0, max 3, IQR 0] vs. 0.0 [min 0, max 3, IQR 0], *p* = 0.04), at 2–3 weeks (median: 0.0 [min 0, max 2, IQR 0] vs. 0.0 [min 0, max 4, IQR 2], *p* < 0.001), at 1–2 months (median: 0.0 [min 0, max 3, IQR 0] vs. 1.0 [min 0, max 4, IQR 2], *p* < 0.001), at 3–4 months (median: 0.0 [min 0, max 4, IQR 0] vs. 1.0 [min 0, max 4, IQR 1], *p* < 0.001), and at 9–12 months (median: 1.0 [min 0, max 3, IQR 2] vs. 2.0 [min 0, max 5, IQR 2], *p* = 0.01) (Table 1).

### 3.3. Rates of Complications and Revisions

Postoperative complications included intraocular pressure (IOP) elevation > 30 mmHg, hyphema, iris obstruction of the stent, rebound iritis, wound leak, choroidal effusions, hypotony maculopathy, corneal decompensation, symptomatic bleb extension, cystoid macular edema, and corneal abrasion. All IOP spikes occurred within the first postoperative month. Complications were observed in 5 of 47 patients (10.6%) in Group 1, which was significantly lower than in Group 2, where 9 of 29 patients (31.0%) experienced complications (*p* = 0.026). Revisions were defined as transconjunctival bleb needling procedures. Bleb needling was performed in 4 of 47 patients (8.5%) in Group 1, compared to 10 of 29 patients (34.5%) in Group 2 (*p* = 0.005).

### 3.4. Kaplan–Meier Survival Analysis

**Figure 1:**
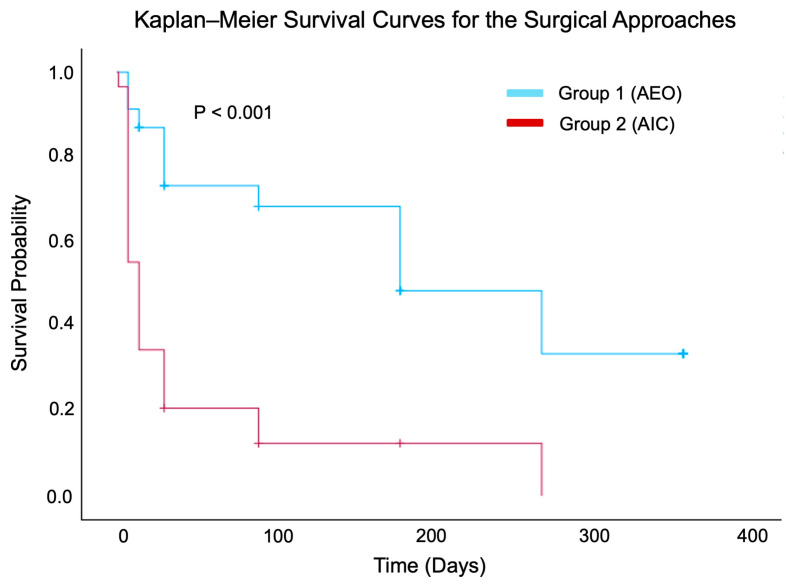
Kaplan–Meier survival curves depicting the time to surgical failure following XEN45 implantation via the AEO and AIC conjunctival approaches. Surgical failure was defined as (1) having an IOP ≤ 5 mmHg or >18 mmHg on two consecutive visits after baseline or (2) the number of IOP-lowering medications being > 0 at any point during the follow-up visits. Censored cases are marked with tick marks. Abbreviations: AEO = ab externo open, AIC = ab interno closed.

Kaplan–Meier analysis demonstrated significantly higher cumulative surgical success in Group 1 (AEO with OCM) compared to Group 2 (AIC) (log-rank test, χ^2^ = 27.150, df = 1, *p* < 0.001) (Figure 1). The mean survival time was 206.5 ± 21.3 days (95% CI: 164.8–248.2) in Group 1 versus 50.4 ± 16.5 days (95% CI: 18.5–83.4) in Group 2. The median survival times were 180 days for Group 1 (95% CI: 98.3–261.7) and 14 days for Group 2 (95% CI: 8.1–19.9). Censoring occurred primarily after 200 days in Group 1 and before 100 days in Group 2. The survival curves showed a pronounced early drop in the closed group, reflecting earlier surgical failure.

## 4. Discussion

In this retrospective case series of patients with refractory open-angle glaucoma, we compared outcomes following XEN45 gel stent implantation using an AEO approach with adjunctive Ologen collagen matrix (Group 1) versus an AIC conjunctival approach (Group 2). Both groups demonstrated meaningful reductions in intraocular pressure (IOP), with no significant differences in IOP reductions between them. However, Group 1 consistently required fewer glaucoma medications at multiple postoperative time points. Additionally, Group 1 demonstrated significantly lower rates of postoperative complications—including IOP spikes and hyphema—as well as fewer surgical revisions. Notably, Kaplan–Meier survival analysis revealed significantly greater cumulative surgical success in the AEO group compared to the AIC group. These findings underscore the safety and efficacy of XGS implantation using the AEO with OCM technique, which was noninferior for IOP control and superior in terms of medication reduction, complication rates, and overall surgical success.

While our study aligns with a similar retrospective study by Tan et al. [16], which did not find a significant difference in IOP reduction between AEO and AIC groups, our results diverge from a recently published study by El Helwe et al. that reported significant differences in postoperative IOP levels, favoring the AEO group [15]. Notably, the discrepancy between our results and those of El Helwe et al. might be attributed to differences in the patient populations. El Helwe et al. included a substantial proportion of mixed-mechanism glaucoma patients in the AEO group, whereas our study primarily focused on open-angle glaucoma cases. This divergence underscores the importance of considering the heterogeneity of glaucoma presentations when evaluating the efficacy of different surgical approaches. Our study further indicates that incorporating OCM into XGS implantation via the AEO approach did not confer superior outcomes in terms of IOP reduction, nor did it enhance the efficacy of this approach. These findings align with those of Park et al., who similarly observed no enhancement in the efficacy of the AEO technique with OCM augmentation for XGS implantation [20].

However, it is noteworthy that although we did not observe a significantly superior reduction in IOP, the AEO with OCM technique resulted in significant medication reductions at multiple time points over the 12-month period. This aligns with our Kaplan–Meier survival analysis, in which surgical failure was defined not only by an IOP outside the target range (≤5 mmHg or >18 mmHg on two consecutive visits) but also by the need for any IOP-lowering medications. Accordingly, the significant survival advantage observed in the AEO group reflects its greater ability to maintain the target IOP without pharmacologic support, despite similar mean IOP values between the groups. These findings are consistent with a study that found a significantly greater medication reduction at 1 year in a group treated using the open-conjunctiva approach regardless of whether the approach was ab externo or ab interno [22]. However, our findings contrast with those of Tan et al., where medication usage was comparable between open and closed techniques. The significant reduction in the number of IOP-lowering medications associated with the open approach in our study could be attributed to several factors. The open approach may facilitate the formation of larger and more functional blebs compared to the closed approach. A well-functioning bleb is essential for maintaining adequate aqueous humor drainage and optimal IOP control. Therefore, patients treated using the open approach may require fewer medications to achieve and maintain target IOP levels. Overall, while the AEO with OCM approach may not have achieved significantly lower IOP levels, it did lead to a reduction in the medication usage required to maintain IOP levels comparable to those in the closed-conjunctiva group. This suggests that the AEO with OCM approach could be more effective than the AIC approach.

Moreover, our study demonstrated the superior performance of the AEO with OCM technique, with significantly lower complication and revision rates as well as greater cumulative surgical success, as shown by the Kaplan–Meier survival analysis. These results are consistent with the findings of Do et al., who demonstrated a comparable success rate and a lower needling rate in an open-conjunctiva group compared to a closed-conjunctiva group [22]. The increased incidences of bleb revisions and stent exposure associated with the closed-conjunctiva approach may be attributed to the technical challenge of consistently and precisely placing the injector needle in the subconjunctival space. Additionally, the higher rate of proximal occlusion of the shunt by the iris observed in closed techniques may stem from the inability to make micro-adjustments once the stent has been deployed. Furthermore, when placing the stent via a closed approach, maintaining the anterior chamber with a cohesive viscoelastic device may artificially widen the angle if the chamber is overfilled or hyperinflated, potentially leading to posterior delivery of the device and subsequent stent obstruction.

However, our findings diverge from those of El Helwe et al., who observed a trend towards a higher rate of postoperative complications in the AEO group, albeit just shy of statistical significance (*p* = 0.06) [15]. Furthermore, they reported a significantly elevated incidence of early hypotony and inflammation in the ab externo open-conjunctiva group compared to the ab externo closed-conjunctiva group, attributing it to increased tissue disruption and the higher likelihood of subclinical microfluidic leaks associated with conjunctival opening. OCM represents one proposed approach to address this issue [23]. Hence, our utilization of OCM may explain the disparity in our findings compared to those of El Helwe et al. Ologen was specifically developed to mechanically prevent conjunctival and episcleral adhesions, thereby reducing bleb fibrosis [17,18]. Our findings suggest that OCM augmentation in the AEO approach may contribute to reduced complication, revision, and failure rates.

This study has several limitations. Firstly, its retrospective design and small sample size may limit the generalizability of the findings. Moreover, the relatively short follow-up period of 12 months, coupled with some patients being lost to follow-up or having irregular follow-up visits, may have introduced potential biases. Additionally, this study’s reliance on outcomes from a single surgeon may not have fully captured variations in surgical technique and expertise across different surgeons. The decision to perform needling at various postoperative time points was left to the discretion of the surgeon, potentially introducing variability in treatment protocols. Further, the inclusion of two different closed-conjunctival techniques in Group 2, namely the standard AIC conjunctiva technique and AIC conjunctiva pneumo-dissection and visco-expansion, may have introduced variability. Due to the small sample size, our study followed the methodology of a prior study that combined these techniques into one group [21]. However, future studies with larger sample sizes should consider analyzing long-term outcomes based on specific surgical techniques.

While a recent study with a substantial sample size reported sustained intraocular pressure reductions and confirmed the long-term safety of XEN45 implantation over a 3-year period, it did not differentiate between the surgical approaches used for stent placement [24]. This highlights the need for further studies comparing outcomes across specific techniques. Additionally, although the overall comparison across all four racial groups did not yield a statistically significant difference (*p* = 0.11), we observed a higher proportion of African American patients in the AIC cohort (76%) compared to the AEO group (55%). This difference, while not statistically significant, may still have clinical relevance. Our study was not powered to evaluate the influence of race on surgical outcomes; however, this observation highlights the need for future studies with larger, more diverse populations to explore potential racial or genetic factors that may impact surgical efficacy. Lastly, while our study utilized an ab externo approach for XGS implantation, it is noteworthy that XGS was originally developed for use with an ab interno approach, necessitating cautious interpretation of our results in this context.

In conclusion, we advocate for the open-conjunctival ab externo technique for XEN45 gel stent implantation with Ologen collagen augmentation. The results of this study indicate a promising trend towards achieving greater rates of complete and qualified surgical success, reduced medication dependency, a lower needling frequency, fewer postoperative complications, and decreased failure rates with this approach. However, to validate these findings and facilitate wider adoption, prospective randomized controlled studies are essential. We envision that this study will catalyze further prospective analyses aimed at enhancing postoperative outcomes for patients with glaucoma, ultimately advancing this field and improving patient care.

## Figures and Tables

**Table 1:** Baseline characteristics.

Parameter	AEO with OCM (Group 1) n = 47	AIC (Group 2) n = 29	*p*-Value
Age at surgery, mean (SD) [years]	68.66 (19.04)	68.97 (10.48)	0.93
Female sex, n (%)	28 (60%)	12 (41%)	0.12
**Race, n (%)**			0.11
African American	26 (55%)	22 (76%)	
Caucasian	15 (32%)	5 (17%)	
Asian	6 (13%)	1 (3%)	
Hispanic or Latino	0 (0%)	1 (3.4%)	
**Glaucoma characteristics**			
Preoperative HVF MD, mean (SD)	−13.67 (7.81)	−12.46 (9.71)	0.72
Preoperative IOP, mean (SD)	24.91 (6.75)	22.76 (7.48)	0.20
Preoperative number of glaucoma medications, median (min–max)	4.00 (2–8)	4.00 (2–5)	0.16
Prior surgery, n (%)	14 (30%)	5 (17%)	.22
**Glaucoma type, n (%)**			0.13
Primary open angle	37 (79%)	28 (97%)	
Mixed mechanism	1 (2%)	0 (0%)	
Pseudoexfoliation	2 (4%)	0 (0%)	
Pigment dispersion	0 (0%)	0 (0%)	
Posner–Schlossman	0 (0%)	1 (3.4%)	
Uveitic	4 (9%)	0 (0%)	
Traumatic open angle	3 (6%)	0 (0%)	

Abbreviations: IOP = intraocular pressure; HVF MD = Humphrey Visual Field Mean Deviation; SD = standard deviation.

**Table 2:** Comparison of mean intraocular pressures and median medication counts at baseline and postoperative intervals across surgical groups.

	AEO, Group 1 (n = 47)		AIC, Group 2 (n = 29)		P (Among Groups)	
	IOP (mmHg)	Meds	IOP (mmHg)	Meds	IOP (mmHg)	Meds
Pre-op	24.92 (23.47–26.36)	4 (2)	22.76 (20.92–24.60)	4 (1)	0.07	0.41
Day 1						
Mean (95% CI)	8.04 (6.57–9.51)	0 (0)	7.40 (5.53–9.26)	0 (0)	0.59	0.21
P (compared with baseline)	<0.001		<0.001		-	-
Week 1						
Mean (95% CI)	9.71 (8.18–11.23)	0 (0)	10.68 (8.79–12.58)	0 (0)	0.43	0.04
P (compared with baseline)	<0.001		<0.001		-	-
Weeks 2–3						
Mean (95% CI)	12.01 (10.36–13.67)	0 (0)	12.54 (10.67–14.40)	0 (2)	0.68	<0.001
P (compared with baseline)	<0.001		<0.001		-	-
Months 1–2						
Mean (95% CI)	12.91 (10.66–13.72)	0 (0)	10.32 (8.43–12.22)	1 (2)	0.13	<0.001
P (compared with baseline)	<0.001		<0.001		-	-
Months 3–4						
Mean (95% CI)	13.24 (11.54–14.94)	0 (0)	11.80 (9.84–13.76)	1 (1)	0.28	<0.001
P (compared with baseline)	<0.001		<0.001		-	-
Months 5–8						
Mean (95% CI)	12.48 (10.55–14.41)	0.5 (2)	11.68 (9.57–13.78)	2 (2)	0.58	0.08
P (compared with baseline)	<0.001		<0.001		-	-
Months 9–12						
Mean (95% CI)	13.87 (11.67–16.07)	1 (2)	12.75 (10.50–15.00)	2 (2)	0.48	0.01
P (compared with baseline)	<0.001		<0.001		-	-

Note: Medication description written as median (IQR). Abbreviations: AEO = ab externo open, AIC = ab interno closed, CI = confidence interval, IOP = intraocular pressure, meds = medications, pre-op = preoperative.

## Data Availability

The datasets presented in this article are not readily available due to U.S. Department of Defense (DoD) restrictions related to research conducted at a military medical center. Requests for data access may be directed to Dr. Won Kim (email: wonkim74@gmail.com) and will be reviewed in accordance with institutional and DoD data sharing policies.

## Supplementary Materials

The following supporting information can be downloaded at: https://www.mdpi.com/article/10.3390/jcm14134426/s1, Table S1: Sample size of patients at each postoperative time point by surgical group.

## Disclaimers

The views expressed herein are those of the authors and do not reflect the official policy or position of the Uniformed Services University of the Health Sciences, Walter Reed National Military Medical Center, San Antonio Military Medical Center, the Department of Defense, or any other agency of the U.S. Government. The identification of specific products or scientific instrumentation is considered an integral part of the scientific endeavor and does not constitute endorsement or implied endorsement on the part of the authors, the DoD, or any component agency. The authors have no proprietary or commercial interests in any materials discussed in this article.

## References

[1] Quigley H.A., Broman A.T. (2006). The number of people with glaucoma worldwide in 2010 and 2020. Br. J. Ophthalmol..

[2] Coleman A.L. (2012). Advances in glaucoma treatment and management: Surgery. Investig. Ophthalmol. Vis. Sci..

[3] Rao A., Cruz R.D. (2022). Trabeculectomy: Does it have a future?. Cureus.

[4] Koike K.J., Chang P.T. (2018). Trabeculectomy: A brief history and review of current trends. Int. Ophthalmol. Clin..

[5] Buffault J., Graber M., Bensmail D., Bluwol É., Jeanteur M.-N., Abitbol O., Benhatchi N., Sauvan L., Lachkar Y. (2020). Efficacy and safety at 6 months of the XEN implant for the management of open angle glaucoma. Sci. Rep..

[6] Galal A., Bilgic A., Eltanamly R., Osman A. (2017). XEN glaucoma implant with mitomycin C 1-year follow-up: Result and complications. J. Ophthalmol..

[7] Grover D.S., Flynn W.J., Bashford K.P., Lewis R.A., Duh Y.-J., Nangia R.S., Niksch B. (2017). Performance and safety of a new ab interno gelatin stent in refractory glaucoma at 12 months. Am. J. Ophthalmol..

[8] Schlenker M.B., Gulamhusein H., Conrad-Hengerer I., Somers A., Lenzhofer M., Stalmans I., Reitsamer H., Hengerer F.H., Ahmed I.I.K. (2017). Efficacy, safety, and risk factors for failure of standalone ab interno gelatin microstent implantation versus standalone trabeculectomy. Ophthalmology.

[9] Birnbaum F.A., Neeson C., Solá-Del Valle D. (2021). Microinvasive glaucoma surgery: An evidence-based review. Semin. Ophthalmol..

[10] Harris J.M., Solá-Del Valle D. (2020). Effective treatment of a normal-tension glaucoma patient with bilateral ab externo XEN Gel Stent implantation. Am. J. Ophthalmol. Case Rep..

[11] Lee R.M., Bouremel Y., Eames I., Brocchini S., Khaw P.T. (2019). The implications of an ab interno versus ab externo surgical approach on outflow resistance of a subconjunctival drainage device for intraocular pressure control. Transl. Vis. Sci. Technol..

[12] Gallardo M.J., Vincent L.R., Porter M. (2022). Comparison of clinical outcomes following gel stent implantation via ab-externo and ab-interno approaches in patients with refractory glaucoma. Clin. Ophthalmol..

[13] Vera V., Gagne S., Myers J.S., Ahmed I.I.K. (2020). Surgical approaches for implanting xen gel stent without conjunctival dissection. Clin. Ophthalmol..

[14] Yuan L., Rana H.S., Lee I., Lai G., Raiciulescu S., Kim W. (2023). Short-term Outcomes of Xen 45 Gel Stent ab Interno Versus ab Externo Transconjunctival Approaches. J. Glaucoma.

[15] El Helwe H., Ingram Z., Neeson C.E., Falah H., Trzcinski J., Lin J.B., Solá-Del Valle D.A. (2024). Comparing Outcomes of 45 Xen Implantation Ab Interno With Closed Conjunctiva to Ab Externo With Open Conjunctiva Approaches. J. Glaucoma.

[16] Tan N.E., Tracer N., Terraciano A., Parikh H.A., Panarelli J.F., Radcliffe N.M. (2021). Comparison of safety and efficacy between ab interno and ab externo approaches to XEN gel stent placement. Clin. Ophthalmol..

[17] Chen H.S.-L., Ritch R., Krupin T., Hsu W.-C. (2006). Control of filtering bleb structure through tissue bioengineering: An animal model. Investig. Ophthalmol. Vis. Sci..

[18] Hsu W.-C., Ritch R., Krupin T., Chen H.S.-L. (2008). Tissue bioengineering for surgical bleb defects: An animal study. Graefe’s Arch. Clin. Exp. Ophthalmol..

[19] Navero-Rodríguez J.M., Espinosa-Barberi G., Morilla-Grasa A., Anton A. (2020). Efficacy of the Ologen collagen matrix in combination with the XEN gel stent implantation in the treatment of open-angle glaucoma: A case-control study. Clin. Exp. Ophthalmol..

[20] Park J., Shin J.W., Sung K.R. (2022). Comparison of surgical outcomes with and without Ologen collagen matrix augmentation during XEN gel stent implantation. BMC Ophthalmol..

[21] Ruda R.C., Yuan L., Lai G.M., Raiciulescu S., Kim W.I. (2023). Clinical outcomes of ab interno placement versus ab externo placement of XEN45 gel stents. Ophthalmol. Glaucoma.

[22] Do A., McGlumphy E., Shukla A., Dangda S., Schuman J.S., Boland M.V., Yohannan J., Panarelli J.F., Craven E.R. (2021). Comparison of clinical outcomes with open versus closed conjunctiva implantation of the XEN45 gel stent. Ophthalmol. Glaucoma.

[23] He M., Wang W., Zhang X., Huang W. (2014). Ologen implant versus mitomycin C for trabeculectomy: A systematic review and meta-analysis. PLoS ONE.

[24] Oddone F., Roberti G., Giammaria S., Posarelli C., Mastropasqua L., Agnifili L., Micelli Ferrari T., Pace V., Sacchi M., Altafini R. (2024). Italian XEN-Glaucoma Treatment Registry (XEN-GTR): Effectiveness and Safety at 36 Months of XEN45 Implant. J. Clin. Med..

