# Supplementary material for: Comparison of XEN45 Gel Stent Outcomes in Glaucoma: Ab Externo Open-Conjunctiva Approach with Ologen vs. Ab Interno Closed-Conjunctiva Approach
Source: J Clin Med. 2025 Jun 21;14(13):4426. doi: 10.3390/jcm14134426 (PMC12249493; doi:10.3390/jcm14134426)
Supplement: Supplementary file 1 [file jcm-14-04426-s001.zip › jcm-3656881-supplementary.pdf]

**Supplementary Materials:**

Table S1. Sample size of patients at each postoperative time point by surgical group.

| Time Point | AEO with OCM (Group 1) | AIC (Group 2) |
|---|---|---|
| Preop | 47 | 29 |
| POD 1 | 45 | 28 |
| POW 1-2 | 41 | 27 |
| POW 2-3 | 36 | 28 |
| POM 1-2 | 43 | 27 |
| POM 3-4 | 35 | 25 |
| POM 5-8 | 26 | 21 |
| POM 9-12 | 23 | 18 |

Abbreviations: AEO = ab interno open, AIC = ab interno closed, OCM = ologen collagen matrix, Preop = Preoperative, POD = Postoperative Day, POW = Postoperative Week, POM = Postoperative Month.
